# Individual host factors and co-infections affect the probability and excretion intensity of endoparasite infections in dairy cows

**DOI:** 10.1186/s13071-025-06974-x

**Published:** 2025-07-30

**Authors:** Anna Sophie Hecker, Marie-Kristin Raulf, Sven König, Katharina May, Christina Strube

**Affiliations:** 1 Institute for Parasitology, Centre for Infection Medicine, University of Veterinary Medicine Hannover, Hannover, Germany; 2 Institute of Animal Breeding and Genetics, Justus-Liebig-University Gießen, Gießen, Germany

**Keywords:** *Calicophoron daubneyi*, Cattle, Coccidia, Faecal egg count, *Fasciola hepatica*, Gastrointestinal nematodes, Liver flukes, Rumen flukes, Strongylidae, Trichostrongyles

## Abstract

**Background:**

Endoparasite infections cause economic losses in dairy farming. Understanding the factors that influence endoparasite prevalence and egg/oocyst excretion is essential for effective parasite control. Environmental and management factors play an important role at herd level; however, factors related to the individual cow, such as the parity number or lactation stage, also contribute to infection. In addition, it is still unclear to what extent co-infections with other endoparasites influence prevalence and egg/oocyst excretion rates.

**Methods:**

Faecal samples from 1,126 cows from 24 dairy herds were copromicroscopically examined for endoparasite infections. Hurdle models were applied to test the effect of parity number, lactation stage and co-infections on the probability and intensity of egg/oocyst excretion of strongyles, *Fasciola hepatica*, rumen flukes and coccidia.

**Results:**

Strongyle eggs were present in all herds and 45.9% of individual cows, *F. hepatica* eggs in 75.0% of herds and 9.9% of cows, rumen fluke eggs in 62.5% of herds and 26.5% of cows and coccidian oocysts in 91.7% of herds and 18.7% of cows. Eggs of *Moniezia* spp., *Trichuris* spp. and *Capillaria* spp. were detected sporadically. Model analysis revealed that the probability of strongyle egg excretion decreased after the first parity, presumably as a result of developing immunity, yet an increase was observed after the fourth parity. With increasing parity number, excretion probability of *F. hepatica* and excretion intensity of rumen flukes increased. Coccidian oocysts excretion was highest in first-parity cows. The lactation stage affected strongyle egg excretion, with the highest probability in early lactation, possibly linked to the negative energy balance in this period. Strongyle co-infections increased the probability of coccidian oocyst excretion (*P* = 0.008), and coccidian co-infections increased both the probability (*P* = 0.011) and intensity (*P* = 0.007) of strongyle egg excretion. Furthermore, coccidian co-infections were associated with a decreased excretion intensity of rumen fluke eggs (*P* = 0.022).

**Conclusions:**

The identification of age groups that are more susceptible to or more likely to spread certain endoparasite taxa and the synergism between strongyle egg and coccidian oocyst excretion can help to implement effective targeted monitoring and control strategies to optimise parasite management in dairy herds.

**Graphical Abstract:**

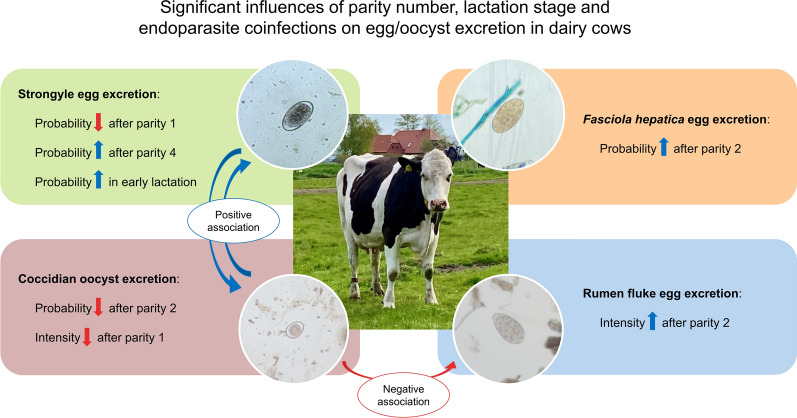

**Supplementary Information:**

The online version contains supplementary material available at 10.1186/s13071-025-06974-x.

## Background

Endoparasite infections are a major concern in dairy production. While most infections are subclinical and often go unnoticed, they can have a substantial economic impact due to reduced milk yield, altered milk composition, decreased carcass weight and impaired fertility [1]. Therefore, endoparasite control is essential not only for cattle health but also for maintaining profitable dairy production. Identifying the factors affecting the prevalence and excretion intensity of endoparasites is crucial for implementing effective treatment and prevention strategies.

Both prevalence and egg excretion are significantly influenced by environmental and management factors [2–4]. While these factors affect the entire herd, individual host factors contribute to varying susceptibility to endoparasite infections among cows. One important factor is the age of the host. For infections with strongyles and *Eimeria* spp., a higher prevalence and an increased numbers of eggs/oocysts per gram of faeces (EPG/OPG) have been observed in heifers and first-parity dairy cows compared with older cows, which acquired immunity after previous exposure to strongyle or coccidia species [2, 5, 6]. By contrast, cattle do not develop a protective immunity against the liver fluke *Fasciola hepatica* and remain susceptible to reinfections throughout their lifespan [7, 8]. Studies investigating associations between host age and rumen fluke infections showed varying results, describing either an increasing prevalence with age [9–11] or no association with age [12, 13].

Another individual host factor that may influence infection susceptibility is the lactation stage. In dairy cows, the early lactation period is characterized by a negative energy balance, which is hypothesised to result in reduced immunity [14, 15]. An increased prevalence of mastitis, metabolic disorders and other clinical diseases has already been reported in the early stage of lactation [14, 16, 17]; however, the influence of the lactation stage has rarely been investigated for endoparasite infections. Perri et al. [18] and May et al. [6] described higher EPGs of gastrointestinal nematodes in early lactation, but data on other endoparasite taxa are lacking.

Most studies investigating the effect of host factors on endoparasite infections have focused on a single endoparasite taxon, without taking into account potentially existing co-infections. Although it has been previously hypothesised that parasites have the ability to exert synergistic or antagonistic effects on each other [19–21], the impact of co-infections on the prevalence and excretion intensity of endoparasites in cattle remains insufficiently studied. A significant positive correlation or association between strongyle and coccidia prevalence and excretion intensity has been reported in cattle [22, 23]. Moreover, positive correlations or interactions between strongyle and *F. hepatica* infections have been described [24, 25], while other studies observed no association between these two taxa [22, 23]. Regarding the interaction between liver and rumen flukes, several studies reported significant positive correlations between *F. hepatica* and rumen fluke EPGs [24, 26], while another study observed a significant negative correlation [27]. So far, no significant associations have been described between infections with coccidia and either *F. hepatica* or rumen flukes [22, 23]. Most studies investigating the impact of co-infections on each other have only considered infections with two endoparasite taxa via simple bivariate analyses, leaving multiple co-infections and their effect on the prevalence and excretion intensity in multivariate models unexplored. However, understanding the interactions between different endoparasite taxa is crucial for developing effective control strategies, for improving dairy cattle health and for maintaining optimal herd productivity.

The objective of this study was to investigate (i) the impact of age and lactation stage as individual host factors and (ii) the impact of endoparasite co-infections on endoparasite excretion probability and intensity (EPG/OPG) for strongyles, *F. hepatica*, rumen flukes and coccidia. A multivariate approach using hurdle models with mixed effects was implemented to separately model effects on excretion probability (binary outcome: infection yes/no) and excretion intensity (EPG/OPG values for positive animals), providing new insights into the role of individual host factors and co-infections on different infection traits.

## Methods

### Faecal sampling and individual cow data

Faecal samples were collected from 1,126 adult dairy cows from 24 herds located in north-western and central Germany (federal states of Lower Saxony, Schleswig–Holstein and Hesse) between May 2021 and February 2022. The sampled herds and cows were selected as part of a project investigating host–parasite interactions between *F. hepatica* and Holstein–Friesian (HF) dairy cows. Thus, parts of this dataset have already been included in studies investigating the in-herd prevalence of liver and rumen flukes [26], the impact of helminth co-infections on milk yield and milk quality [24] and heritabilities for liver and rumen fluke infection traits [28].

Herd selection was based on suspected liver fluke infections, either due to high bulk tank milk antibody levels or reports of infected livers from the abattoir. Cows had access to pasture in all dairy herds. Anthelmintic treatment of dry cows was implemented in 66.7% (16/24) of herds, but data on treatments of individual cows were not available. From each herd, rectal faecal samples were collected once of approximately 50 randomly selected cows. The breed composition was 78.7% (886/1126) black and white HF, 6.1% (69/1126) Black Pied dual-purpose (German: *Deutsches Schwarzbuntes Niederungsrind*), 4.4% (50/1126) dairy × beef crosses, 3.7% (42/1126) dairy × dairy crosses, 2.8% (32/1126) Angler, 2.7% (30/1126) red and white HF and 1.5% (17/1126) unknown breeds. Individual cow data (e.g. parity number and lactation stage) were provided by the National Genetic Evaluation Centre (*Vereinigte Informationssysteme Tierhaltung w.V.*, VIT). Animal numbers per parity were 337 cows in parity 1, 282 cows in parity 2, 207 cows in parity 3, 121 cows in parity 4, 78 cows in parity 5 and 101 cows in parity ≥ 6. The maximum recorded parity number was 12. However, only 11 cows were in parity > 8.

### Copromicroscopical examinations

Each faecal sample was screened for endoparasite eggs, oocysts and larvae using three different techniques. To check for *Dictyocaulus viviparus* larvae, the Baermann method was used. The Baermann funnels were loaded with approximately 10–15 g of fresh faeces on the day of collection and the larvae allowed to migrate for 12–18 h. Samples were examined with a transmitted-light microscope (Axiostar plus, Carl Zeiss, Oberkochen, Germany) at 50× magnification. For flotation, the FLOTAC-100 apparatus was used applying the double technique with 10 g of faeces according to published instructions [29]. Zinc sulphate was used as flotation solution (specific gravity: 1.30). Eggs and oocysts were counted under the transmitted-light microscope (Axiostar plus, Carl Zeiss, Oberkochen, Germany) at 100× magnification, and the number of eggs/oocysts was multiplied by two to obtain EPG/OPG values [29]. For the detection of trematode eggs, a classical sedimentation technique was conducted [30] but was modified by the use of 10 g of faeces, as described by Hecker et al. [26]. After three to four sedimentation steps, the obtained sediment was mixed with one drop of 1% methylene blue (Merck, Darmstadt, Germany) and examined with the transmitted-light microscope (Axiostar plus, Carl Zeiss, Oberkochen, Germany) at 50× magnification. Due to the use of 10 g of faeces, egg counts were divided by ten to calculate EPGs.

### Statistical analyses

All descriptive statistics (means, standard deviations and confidence intervals) and model analyses were conducted using R version 4.4.0 [31]. Two-part hurdle models with mixed effects were used to model egg/oocyst excretion probability and intensity for strongyles, *F. hepatica*, rumen flukes and coccidia. Hurdle models were fitted for each endoparasite separately using the R packages glmmTMB [32] and GLMMadaptive [33]. The hurdle models consist of a binary model, which models the binary defined excretion probability (excretion yes/no), while a zero-truncated count model using a negative binomial distribution simulates excretion intensity by modelling EPG/OPG values of the positive cows. Positive estimates indicate a higher egg excretion probability in the binary model and higher EPG/OPG values in the count model. To find the best-fitting model, all models within each endoparasite taxon were evaluated by comparing the Akaike information criterion (AIC) and residual plots generated by the DHARMa package [34].

The dataset for each model only included cows from herds where the respective endoparasite taxon was present, i.e. cows from all 24 herds were included in the strongyle model (*n* = 1,126), cows from 18 herds in the *F. hepatica* model (*n* = 898), cows from 15 herds in the rumen fluke model (*n* = 698) and cows from 22 herds in the coccidia model (*n* = 1,028). Fixed effects included parity number (six classes: 1, 2, 3, 4, 5 and ≥ 6) and co-infection with strongyles, *F. hepatica*, rumen flukes and coccidia, each as a binary variable (infected yes/no). For example, in the strongyle model, infection status for *F. hepatica*, rumen flukes and coccidia were included in the model, but as three separate fixed effects. The inclusion of the co-infection status as a binary variable resulted in better model fits than modelling EPGs/OPGs as a covariate. The lactation stage (days in milk, DIM) was modelled as a covariate. Herd and breed were included as random factors. Other factors tested in the models were sampling month and anthelmintic treatment (yes/no) in the herd. However, these factors were excluded owing to a lack of improvement in model fit. All models were compared with a null model containing only the random factors using a likelihood ratio test. All *P*-values ≤ 0.05 were considered statistically significant.

## Results

### In-herd prevalence and co-infections on herd-level

Results of the copromicroscopical examinations are summarised in Table 1, which displays in-herd prevalence rates and mean EPG/OPG values in each herd for each parasite taxon. Strongyle eggs were observed in all 24 herds. Eggs of *F. hepatica* were detected in 75.0% (18/24) of herds, rumen fluke eggs in 62.5% (15/24) of herds and coccidian oocysts in 91.7% (22/24) of herds. Eggs of *Moniezia* spp., *Trichuris* spp. and *Capillaria* spp. were identified in 37.5% (9/24), 8.3% (2/24) and 4.2% (1/24) of herds, respectively.

**Table 1:** Copromicroscopic examination results of 24 German dairy herds

Herd	Strongyles		*F. hepatica*		Rumen flukes		Coccidia		*Moniezia* spp.		*Trichuris* spp.		*Capillaria* spp.	
	In-herd prevalence	Mean EPG (SD)	In-herd prevalence	Mean EPG (SD)	In-herd prevalence	Mean EPG (SD)	In-herd prevalence	Mean OPG (SD)	In-herd prevalence	Mean EPG (SD)	In-herd prevalence	Mean EPG (SD)	In-herd prevalence	Mean EPG (SD)
1	75.00% (27/36)	3.19 (± 2.24)	5.56% (2/36)	0.20 (± 0.14)	− (0/36)	–	11.11% (4/36)	2.50 (± 1.00)	2.78% (1/36)	6.00 (n.d.^a^)	− (0/36)	–	− (0/36)	–
2	84.78% (39/46)	9.28 (± 13.8)	36.96% (17/46)	0.21 (± 0.16)	34.78% (16/46)	0.31 (± 0.28)	28.26% (13/46)	5.23 (± 5.45)	− (0/46)	–	− (0/46)	–	− (0/46)	–
3	68.00% (34/50)	6.65 (± 6.40)	36.00% (18/50)	0.69 (± 0.72)	− (0/50)	–	40.00% (20/50)	16.10 (± 42.38)	8.00% (4/50)	8.50 (± 10.38)	− (0/50)	–	− (0/50)	–
4	40.43% (19/47)	6.63 (± 5.46)	− (0/47)	–	− (0/47)	–	8.51% (4/47)	4.50 (± 3.79)	− (0/47)	–	− (0/47)	–	4.26% (2/47)	2.00 (± 0.00)
5	46.94% (23/49)	4.35 (± 2.93)	2.04% (1/49)	0.10 (n.d.)	− (0/49)	–	− (0/49)	–	− (0/49)	–	− (0/49)	–	− (0/49)	–
6	18.75% (9/48)	2.67 (± 1.41)	2.08% (1/48)	0.10 (n.d.)	− (0/48)	–	4.17% (2/48)	2.00 (± 0.00)	− (0/48)	–	− (0/48)	–	− (0/48)	–
7	52.08% (25/48)	6.40 (± 6.56)	12.50% (6/48)	0.30 (± 0.24)	4.17% (2/48)	1.60 (± 2.12)	45.83% (22/48)	7.36 (± 7.82)	− (0/48)	–	− (0/48)	–	− (0/48)	–
8	20.00% (10/50)	7.20 (± 6.41)	8.00% (4/50)	0.20 (± 0.14)	− (0/50)	–	4.00% (2/50)	2.00 (± 0.00)	− (0/50)	–	− (0/50)	–	− (0/50)	–
9	44.90% (22/49)	10.36 (± 28.34)	20.41% (10/49)	0.43 (± 0.36)	4.08% (2/49)	0.15 (± 0.07)	10.20% (5/49)	8.80 (± 9.01)	− (0/49)	–	− (0/49)	–	− (0/49)	–
10	36.73% (18/49)	8.78 (± 12.27)	− (0/49)	–	− (0/49)	–	22.45% (11/49)	6.55 (± 7.43)	− (0/49)	–	− (0/49)	–	− (0/49)	–
11	39.13% (18/46)	5.22 (± 5.09)	10.87% (5/46)	0.20 (± 0.22)	13.04% (6/46)	0.15 (± 0.05)	15.22% (7/46)	3.14 (± 1.57)	6.52% (3/46)	6.00 (± 3.46)	2.17% (1/46)	2.00 (n.d.)	− (0/46)	–
12	72.34% (34/47)	7.29 (± 5.46)	6.38% (3/47)	0.17 (± 0.06)	93.63% (44/47)	6.13 (± 7.92)	17.02% (8/47)	7.00 (± 7.01)	2.13% (1/47)	84.00 (n.d.)	− (0/47)	–	− (0/47)	–
13	56.00% (28/50)	10.75 (± 15.14)	− (0/50)	–	6.00% (3/50)	0.20 (± 0.17)	8.00% (4/50)	3.00 (± 1.15)	− (0/50)	–	− (0/50)	–	− (0/50)	–
14	75.00% (30/40)	6.20 (± 7.15)	15.00% (6/40)	0.40 (± 0.37)	75.00% (30/40)	2.89 (± 6.31)	35.00% (14/40)	2.71 (± 1.49)	5.00% (2/40)	8.00 (± 5.66)	− (0/40)	–	− (0/40)	–
15	54.17% (26/48)	9.54 (± 12.47)	27.08% (13/48)	0.18 (± 0.14)	100% (48/48)	57.04 (± 64.63)	39.58% (19/48)	6.32 (± 5.90)	− (0/48)	–	− (0/48)	–	− (0/48)	–
16	57.14% (28/49)	5.14 (± 4.91)	10.20% (5/49)	0.24 (± 0.26)	− (0/49)	–	34.69% (17/49)	9.06 (± 7.88)	− (0/49)	–	− (0/49)	–	− (0/49)	–
17	72.34% (34/47)	5.17 (± 5.02)	7.69% (4/52)	0.10 (± 0.00)	100% (52/52)	33.83 (± 42.85)	5.77% (3/52)	2.00 (± 0.00)	− (0/52)	–	− (0/52)	–	− (0/52)	–
18	26.67% (12/45)	7.17 (± 7.31)	13.33% (6/45)	0.18 (± 0.16)	80.00% (36/45)	7.36 (± 9.58)	22.22% (10/45)	13.20 (± 16.01)	− (0/45)	–	− (0/45)	–	− (0/45)	–
19	10.87% (5/46)	4.00 (± 2.00)	2.17% (1/46)	0.20 (n.d.)	2.17% (1/46)	0.10 (n.d.)	17.39% (8/46)	2.75 (± 1.04)	− (0/46)	–	− (0/46)	–	− (0/46)	–
20	31.25% (10/32)	4.40 (± 3.24)	− (0/32)	–	78.13% (25/32)	17.91 (± 28.73)	46.88% (15/32)	5.20 (± 3.61)	9.38 (3/32)	6.00 (± 4.00)	− (0/32)	–	− (0/32)	–
21	2.00% (1/50)	2.00 (n.d.)	− (0/50)	–	12.00% (6/50)	5.72 (± 13.08)	6.00% (3/50)	2.67 (± 1.15)	2.00 (1/50)	6.00 (n.d.)	− (0/50)	–	− (0/50)	–
22	38.00% (19/50)	2.63 (± 1.16)	− (0/50)	–	− (0/50)	–	26.00% (13/50)	8.15 (± 8.31)	− (0/50)	–	2.00% (1/50)	2.00 (n.d.)	− (0/50)	–
23	32.65% (16/49)	4.50 (± 5.03)	4.08% (2/49)	0.25 (± 0.07)	14.29% (7/49)	3.29 (± 4.64)	− (0/49)	–	2.04% (1/49)	12.00 (n.d.)	− (0/49)	–	− (0/49)	–
24	60.00% (30/50)	4.73 (± 4.65)	14.00% (7/50)	0.19 (± 0.15)	40.00% (20/50)	0.80 (± 1.25)	14.00% (7/50)	3.14 (± 3.02)	8.00% (4/50)	7.50 (± 7.00)	− (0/50)	–	− (0/50)	–
Mean (SD)	46.18% (± 21.26)	6.01 (± 2.46)	13.02% (± 10.44)	0.24 (± 0.14)	43.82% (± 37.85)	9.17 (± 16.05)	21.01% (± 13.71)	5.61 (± 3.76)	5.09% (± 2.80)	16.00 (± 25.57)	2.09% (± 0.09)	2.00 (± 0.00)	4.26% (n.d.)	2.00 (n.d.)

For each endoparasite taxon, in-herd prevalence (with the number of positive/sampled animals in parenthesis) and mean EPG/OPG values of positive cows including standard deviation of the mean (SD) are shown. For improved clarity, in-herd prevalence and mean EPG of negative herds were marked with a minus symbol instead of ‘0.00’

^a^*n.d.* not determined

All 24 herds harboured multiple endoparasite taxa. Co-infections with two different endoparasite taxa were detected in 8.3% (2/24) of herds, with three different taxa in 25.0% (6/24) of herds, with four taxa in 50.0% (12/24) of herds and with five taxa in 12.5% (3/24) of herds, and one herd (4.2% [1/24]) harboured six different endoparasite taxa. Considering only the four most prevalent taxa, coninfections with strongyles and coccidia were detected in 12.5% (3/24) of herds, while one herd (4.2% [1/24]) was co-infected with strongyles and *F. hepatica*. In the remaining herds (83.3% [20/24)], at least three of the four most common endoparasite taxa were present. Strongyles, *F. hepatica* and coccidia were detected in 20.8% (5/24) of herds, while 12.5% (3/24) of herds were positive for strongyles, rumen flukes and coccidia. One herd (4.2% [1/24]) harboured strongyles, *F. hepatica* and rumen flukes. All four endoparasite taxa, i.e. strongyles, *F. hepatica*, rumen flukes and coccidia, were detected in 45.8% (11/24) of herds.

### Endoparasite prevalence and co-infections in individual cows

In total, 65.8% (741/1126) of cows excreted endoparasite eggs or oocysts. Strongyle eggs were excreted by 45.9% (517/1126) of cows with a mean EPG of 6.5 (95% CI: 5.7–7.4 EPG, range: 2.0–134.0 EPG), and 9.9% (111/1126) of cows were positive for *F. hepatica* eggs with a mean EPG of 0.3 (95%CI: 0.2–0.4 EPG, range: 0.1–2.5 EPG). Rumen fluke eggs were detected in 26.5% (298/1126) of cows with a mean EPG of 19.0 (95% CI: 14.6–23.3 EPG, range: 0.1–292.4 EPG). Coccidian oocysts were excreted by 18.7% (211/1126) of cows with a mean OPG of 7.0 (95% CI: 5.0–9.0 OPG, range: 2.0–194.0 OPG). *Moniezia* spp. eggs were observed in 1.8% (20/1126) of cows with a mean EPG of 13.0 (95% CI: 4.3–21.7 EPG, range: 2.0–84.0 EPG), *Trichuris* spp. eggs in 0.2% (2/1126) of cows with a mean EPG of 2.0 (95% CI: n.d., range: 2.0–2.0 EPG) and *Capillaria* spp. in 0.2% (2/1126) of cows with a mean EPG of 2.0 (95% CI: n.d., range: 2.0–2.0 EPG).

Mono-infections were detected in 36.6% (412/1126) of cows, while 22.1% (249/1126) of cows were co-infected with two endoparasite taxa, 6.1% (69/1126) of cows with three and 1.0% (11/1126) of cows with four taxa. Considering only the four most prevalent taxa, prevalence and EPG/OPG values for all potential co-infection combinations are displayed in Table 2.

**Table 2:** Pre-model comparison of the of co-infection prevalence in individual cows (with the number of positive/sampled animals in parenthesis) and mean EPG/OPG values including the standard deviation of the mean (SD) of mono- and co-infected cows from 24 German dairy herds

		Strongyles		*F. hepatica*		Rumen flukes		Coccidia	
		Prevalence	Mean EPG (SD)	Prevalence	Mean EPG (SD)	Prevalence	Mean EPG (SD)	Prevalence	Mean OPG (SD)
Mono-infected		21.49% (242/1126)	5.91 (± 11.05)	2.34% (27/1126)	0.40 (± 0.50)	8.26% (93/1126)	22.52 (± 42.21)	5.24% (59/1126)	8.20 (± 25.12)
Co-infected (total)		24.42% (275/1126)	7.06 (± 8.45)	7.46% (84/1126)	0.28 (± 0.34)	18.21% (205/1126)	17.34 (± 36.58)	13.50% (152/1126)	6.55 (± 7.56)
Co-infected with one other taxon		18.29% (206/1126)	6.99 (± 8.90)	4.53% (51/1126)	0.30 (± 0.40)	12.08% (136/1126)	20.31 (± 42.06)	8.26% (93/1126)	7.51 (± 8.48)
	Strongyles	–	–	2.84% (32/1126)	0.40 (± 0.48)	9.24% (104/1126)	21.49 (± 46.52)	6.22% (70/1126)	8.14 (± 8.75)
	*F. hepatica*	2.84% (32/1126)	4.62 (± 4.20)	–	–	1.24% (14/1126)	20.99 (± 28.39)	0.44% (5/1126)	3.60 (± 1.67)
	Rumen flukes	9.24% (104/1126)	6.65 (± 7.59)	1.24% (14/1126)	0.14 (± 0.05)	–	–	1.60% (18/1126)	6.11 (± 8.33)
	Coccidia	6.22% (70/1126)	8.57 (± 11.66)	0.44% (5/1126)	0.12 (± 0.04)	1.60% (18/1126)	13.02 (± 15.89)	–	–
Co-infected with two other taxa		5.15% (58/1126)	6.69 (± 5.69)	1.95% (22/1126)	0.25 (± 0.19)	5.15% (58/1126)	10.58 (± 20.94)	4.26% (48/1126)	5.25 (± 5.92)
	Strongyles and* F. hepatica*	–	–	–	–	1.24% (14/1126)	13.06 (± 24.1)	0.36% (4/1126)	6.00 (± 6.00)
	Strongyles and rumen flukes	–	–	1.24% (14/1126)	0.28 (± 0.21)	–	–	3.55% (40/1126)	4.90 (± 6.13)
	Strongyles and coccidia	–	–	0.36% (4/1126)	0.30 (± 0.16)	3.55% (40/1126)	5.70 (± 11.74)	–	–
	*F. hepatica* and rumen flukes	1.24% (14/1126)	4.71 (± 2.79)	–	–	–	–	0.36% (4/1126)	8.00 (± 4.32)
	*F. hepatica* and coccidia	0.36% (4/1126)	9.00 (± 11.37)	–	–	0.36% (4/1126)	50.68 (± 39.16)	–	–
	Rumen flukes and coccidia	3.55% (40/1126)	7.15 (± 5.71)	0.36% (4/1126)	0.12 (± 0.05)	–	–	–	–
Co-infected with all three other taxa		0.98% (11/1126)	10.36 (± 11.62)	0.98% (11/1126)	0.24 (± 0.27)	0.98% (11/1126)	16.22 (± 23.06)	0.98% (11/1126)	4.18 (± 3.84)
Total		45.91% (517/1126)	6.52 (± 9.76)	9.86% (111/1126)	0.31 (± 0.39)	26.47% (298/1126)	18.96 (± 38.43)	18.74% (211/1126)	7.01 (± 14.69)

### Impact of parity number and lactation stage on endoparasite infections

**Fig. 1:**
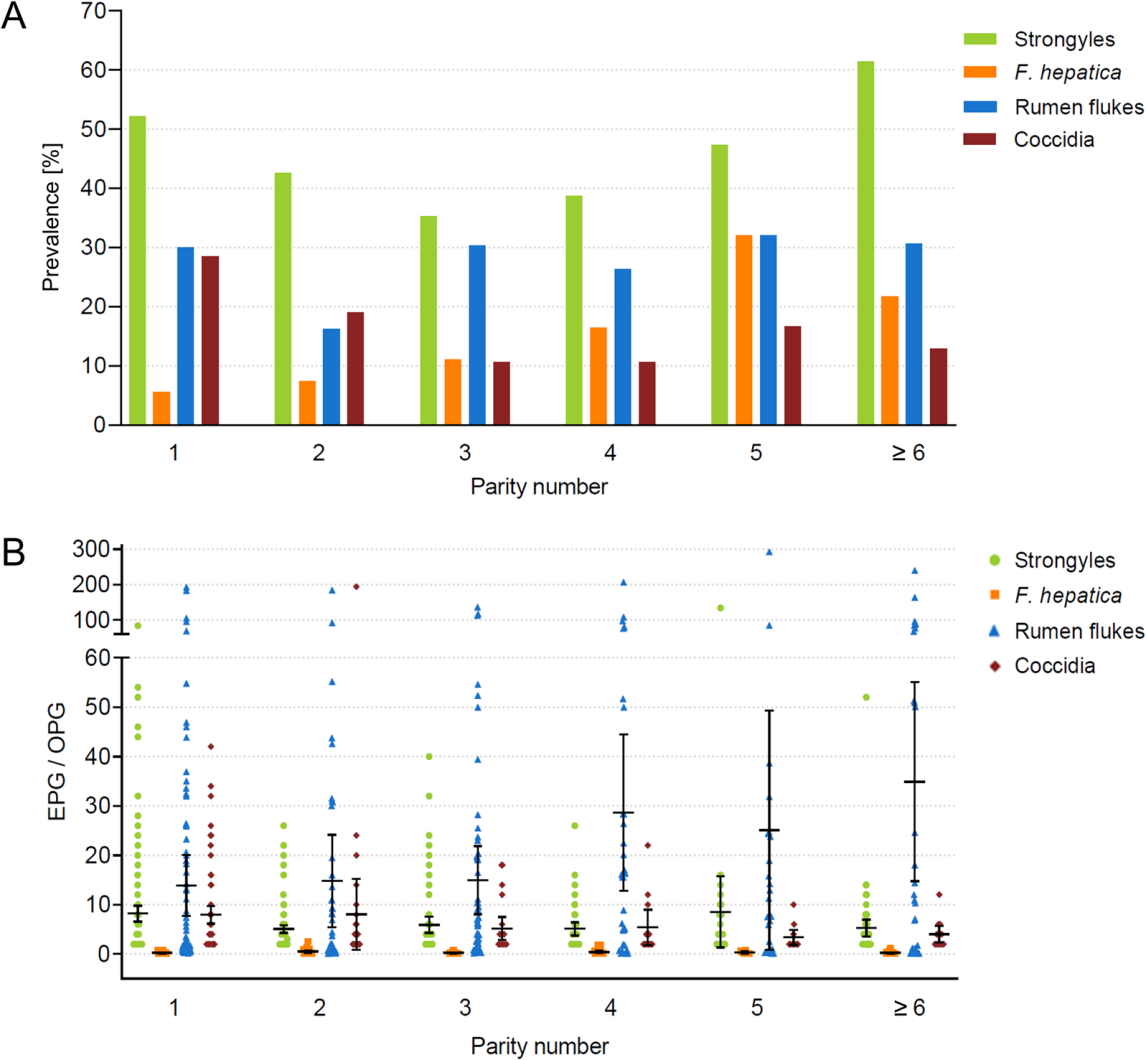
Pre-model display of the copromicroscopic examination results of 1,126 dairy cows from 24 farms in Germany, categorised by parity number. The bar plot shows the prevalence, i.e. the percentage of positive cows for each endoparasite taxon (**A**). The scatter plot illustrates the intensity of egg/oocyst excretion (EPG/OPG of infected cows) with the mean marked as lines and 95% confidence intervals as bars (**B**)

Pre-model comparisons of endoparasite prevalence and EPG/OPG values for each parity number are displayed in Table 3 and Fig. 1, which illustrate a variation in prevalence and egg/oocyst counts between parities without considering other influencing factors.

**Table 3:** Pre-model comparison of endoparasite prevalence (with the number of positive/sampled animals in parenthesis) and mean EPG/OPG values including the standard deviation of the mean (SD) of infected cows in different parity numbers from 24 German dairy herds

Parity number	Strongyles		*F. hepatica*		Rumen flukes		Coccidia	
	Prevalence	Mean EPG (SD)	Prevalence	Mean EPG (SD)	Prevalence	Mean EPG (SD)	Prevalence	Mean OPG (SD)
1	52.82% (178/337)	8.18 (± 11.06)	5.64% (19/337)	0.22 (± 0.20)	29.97% (101/337)	13.90 (± 31.24)	28.49% (96/337)	7.98 (± 8.78)
2	42.55% (120/282)	5.04 (± 4.40)	7.45% (21/282)	0.48 (± 0.58)	16.31% (46/282)	14.78 (± 31.48)	19.15% (54/282)	8.04 (± 26.18)
3	35.27% (73/207)	5.89 (± 7.06)	11.11% (23/207)	0.21 (± 0.20)	30.43% (63/207)	14.93 (± 27.45)	10.63% (22/207)	5.18 (± 5.22)
4	38.84% (47/121)	5.11 (± 4.58)	16.53% (20/121)	0.40 (± 0.52)	26.45% (32/121)	28.63 (± 44.00)	10.74% (13/121)	5.38 (± 5.97)
5	47.44% (37/78)	8.54 (± 21.53)	35.90% (25/78)	0.35 (± 0.26)	35.90% (25/78)	25.09 (± 58.63)	16.67% (13/78)	3.38 (± 2.50)
≥ 6	61.39% (62/101)	5.26 (± 6.83)	21.78% (22/101)	0.24 (± 0.26)	30.69% (31/101)	34.90 (± 55.06)	12.87% (13/101)	4.00 (± 2.83)

**Fig. 2:**
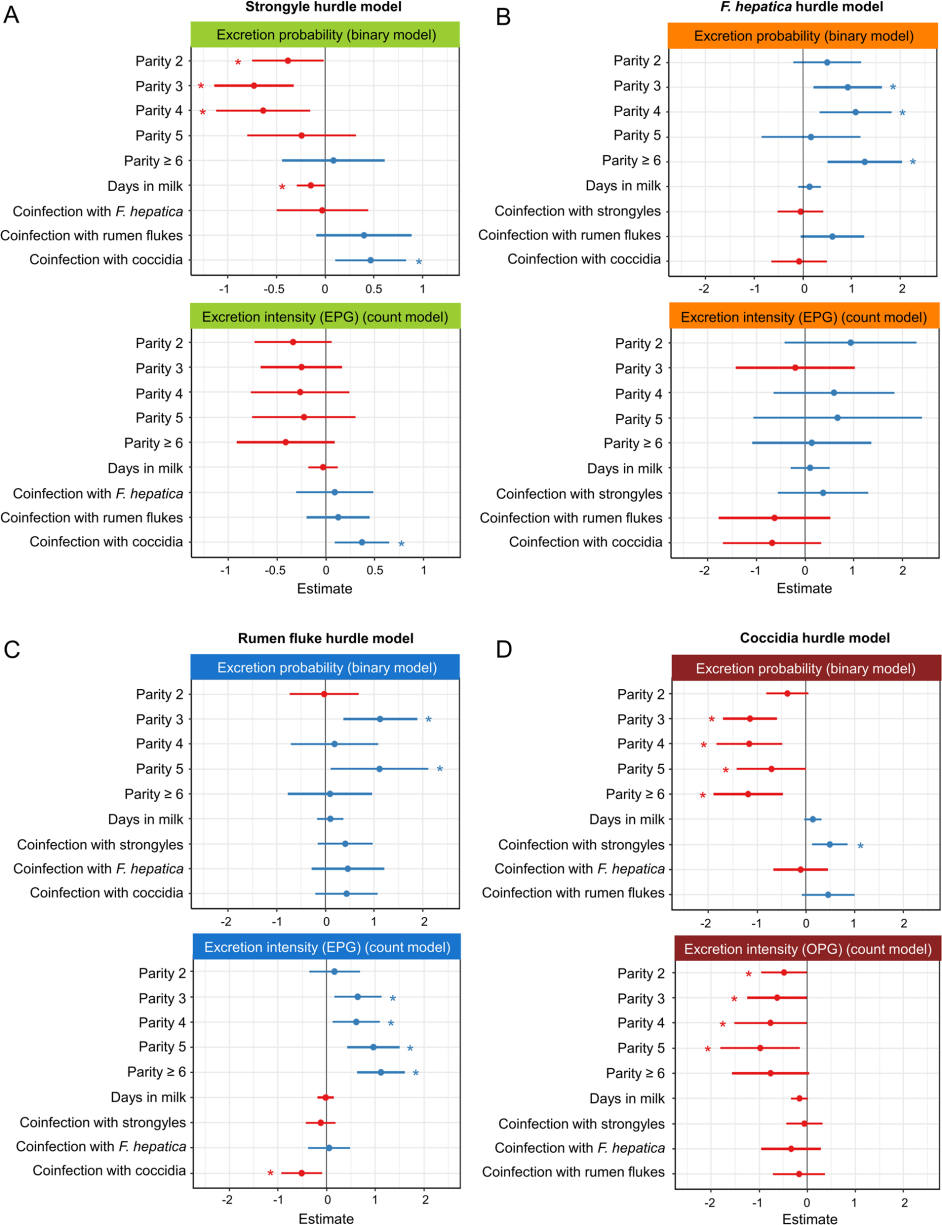
Estimate plots of the hurdle models to visualise the influence of the fixed effects on endoparasite egg/oocyst excretion of German dairy cows for strongyle eggs (**A**), *F. hepatica* eggs (**B**), rumen fluke eggs (**C**) and coccidian oocysts (**D**). The upper plot represents the binary model (excretion probability), and the lower plot shows the results of the zero-truncated count model (excretion intensity of infected animals). Standardised estimates with their 95% confidence intervals depicted as bars are displayed. Positive estimates are displayed in blue (increase of the excretion probability/intensity), while negative estimates are coloured in red (decrease of the excretion probability/intensity). Statistically significant estimates (*P* ≤ 0.05) are marked with an asterisk. Variables for parities 2 to ≥ 6 are to be interpreted in relation to parity 1

Model results revealed that the parity number had a substantial impact on all endoparasite taxa. In the strongyle model, parity number significantly influenced excretion probability (*P* = 0.002), as indicated by the binary model, but did not impact excretion intensity (EPG) (*P* = 0.550), as shown in the count model (Fig. 2A; Additional file 1: Table S1). The binary model revealed a reduced statistical probability of strongyle egg excretion as indicated by a negative estimate for cows in parities 2 (*P* = 0.038), 3 (*P* < 0.001) and 4 (*P* = 0.010) compared with first-parity cows. In cows of parity ≥ 5, the probability of excretion approached that of first-parity cows. For *F. hepatica*, parity number significantly influenced the excretion probability (*P* = 0.006) but not the excretion intensity (*P* = 0.308) (Fig. 2B, Additional file 1: Table S2). The estimates from the binary model were positive for all parities ≥ 2, with estimates for parities 3, 4 and ≥ 6 being significantly higher compared with parity 1 (*P* = 0.009, *P* = 0.004 and *P* < 0.001, respectively), indicating a higher statistical probability of *F. hepatica* egg excretion as of parity 2. Rumen fluke egg excretion probability and intensity were both influenced by parity number (*P* = 0.016 and *P* = 0.001, respectively) (Fig. 2C, Additional file 1: Table S3). Excretion probability was significantly higher in parities 3 and 5 compared with parity 1 (*P* = 0.004 and *P* = 0.029, respectively), and excretion intensity increased significantly from parity 3 onwards compared with the first parity (*P* = 0.009 for parity 3, *P* = 0.013 for parity 4, *P* < 0.001 for parity 5, *P* < 0.001 for parity ≥ 6), indicating higher EPGs with advancing age. The excretion probability and intensity of coccidian oocysts was negatively influenced by parity number (*P* < 0.001 and *P* = 0.030, respectively) (Fig. 2D, Additional file 1: Table S4). Excretion probability decreased significantly from parity 3 onwards compared with parity 1 (*P* < 0.001 for parity 3, *P* = 0.001 for parity 4, *P* = 0.049 for parity 5 and *P* = 0.001 for parity ≥ 6), and significantly lower excretion intensities were observed in parities 2 (*P* = 0.043), 3 (*P* = 0.046), 4 (*P* = 0.046) and 5 (*P* = 0.019) compared with first-parity cows.

Days in milk had a significant negative impact on the excretion probability of strongyle eggs (*P* = 0.037), indicating a declining probability of egg excretion throughout lactation (Fig. 2A, Additional file 1: Table S1). However, the lactation stage had neither an influence on the excretion intensity of strongyle eggs nor on the excretion probability or intensity of the other endoparasites tested (Fig. 2, Additional file 1: Tables S1–4).

### Impact of co-infection status on endoparasite infections

**Fig. 3:**
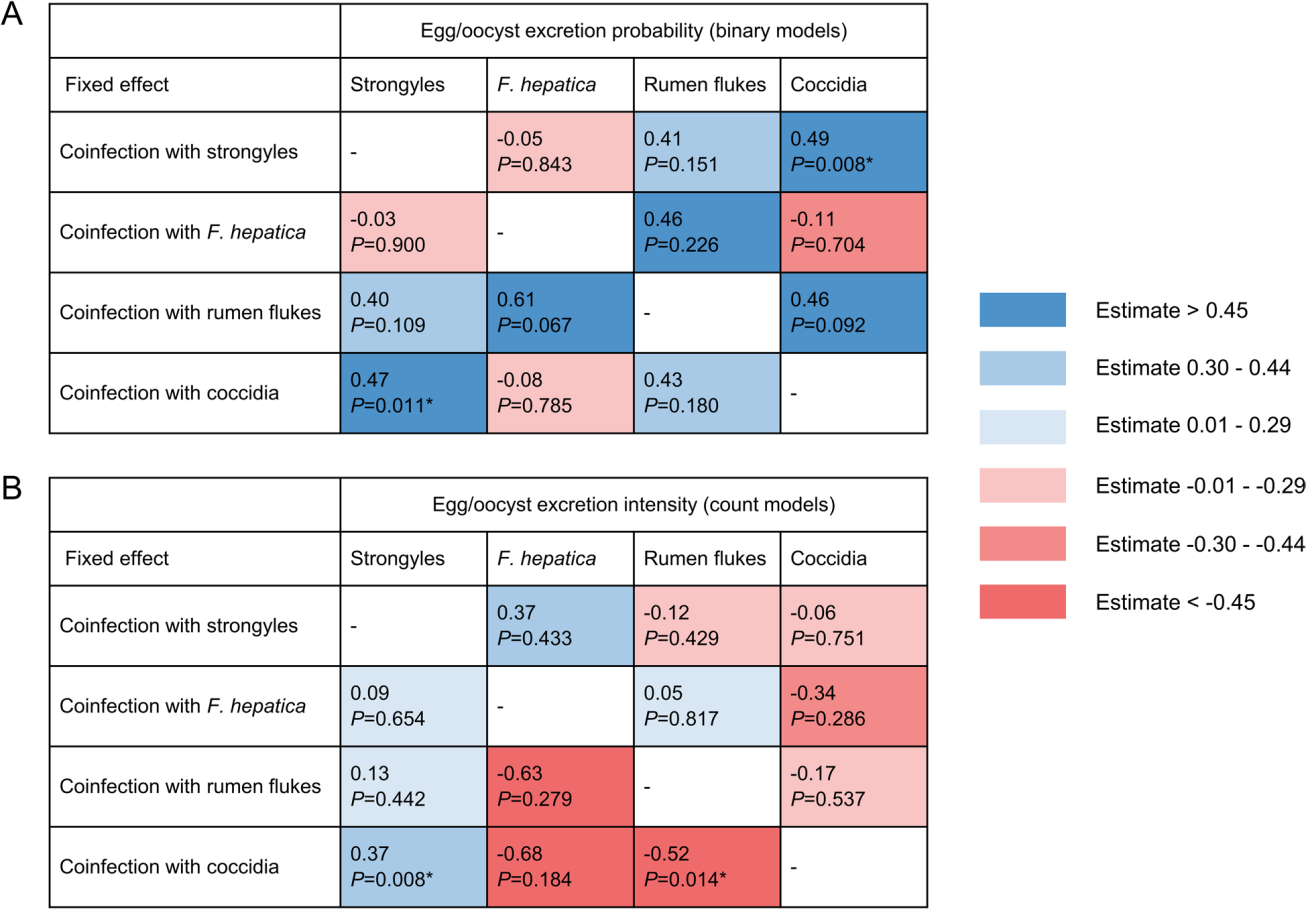
Compilation of the estimated effects of co-infections on endoparasite egg/oocyst excretion probability (**A**) and excretion intensity (**B**) from all hurdle models. A heat map illustrates the estimates, with positive estimates depicted in shades of blue and negative estimates in shades of red. Statistically significant estimates (*P* ≤ 0.05) are marked with an asterisk

The estimated influence of co-infections in each model is depicted in Fig. 2A–D, and a summary of their effects across all models is provided in Fig. 3. Strongyle egg excretion was significantly influenced by a co-infection with coccidia. Both excretion probability (*P* = 0.011) and excretion intensity (*P* = 0.008) of strongyle eggs were higher in cows co-infected with coccidia, while other co-infections did not affect strongyle egg excretion. The excretion of *F. hepatica* eggs was not significantly influenced by any of the co-infections tested. Rumen fluke EPGs were significantly lower in cows co-infected with coccidia (*P* = 0.014), although the probability of egg excretion was unaffected. Co-infections with strongyles or *F. hepatica* did not influence rumen fluke egg excretion. The excretion probability of coccidian oocysts was higher in cows co-infected with strongyles (*P* = 0.008), but excretion intensity was not affected. Co-infections with *F. hepatica* or rumen flukes had no significant effect on oocyst excretion probability or intensity.

## Discussion

In this study, the impact of individual host factors and co-infections on the most prevalent endoparasite taxa in German dairy cows, i.e. strongyles, *F. hepatica*, rumen flukes and coccidia, was analysed. The parity number had a pronounced influence on egg/oocyst excretion of these endoparasite taxa. For strongyle infections, egg excretion probability decreased significantly after the first parity. After the fourth parity, excretion probability returned to the level of first-parity cows. The higher infection probability in first-parity cows aligns with findings from previous studies [35, 36]. Cattle are known to develop an immunity against most gastrointestinal nematodes within or after the first grazing season [35]. Against *Ostertagia ostertagi*, the most frequent strongyle species in cattle, immunity becomes evident only in cows older than 2 years following a prolonged period of exposure [35], which explains the apparent decline in excretion probability after the first parity. The recurring increase in excretion probability in older cows may be attributed to a decline in immunological capacity. Previous studies have shown that immune function diminishes with advancing age, as indicated by higher incidences of infectious diseases, reduced leucocyte counts and adverse shifts in the gut microbiome in older cows [37–39]. Strongyle EPGs were not significantly affected by parity number in this study, which is in contrast to previous observations indicating a decline in egg counts with increasing parity [6, 35]. However, the overall low EPG levels detected in this study may have hindered the identification of a significant decline.

For *F. hepatica*, the model revealed a significant increase in egg excretion probability with higher parity. Cattle do not develop protective immunity against *F. hepatica* and remain susceptible to reinfections throughout their lives [40]. The continuous exposure to (re-)infections on contaminated pastures, along with the long life span of liver flukes in cattle, which can reach up to 26 months [41], is an explanation for the increasing excretion probability with age. Another reason may be the reduced immune capacity associated with increasing parity [37, 38], as discussed above. Consistent with this, da Costa et al. [42] also noted a significant increase in *F. hepatica* prevalence with age in cattle. The decreased probability in parity 5 may be a statistical inaccuracy, as the sample size was lowest in this parity. Interestingly, the parity number had no significant influence on *F. hepatica* EPGs, although lower worm burdens and egg counts in older cattle have previously been reported [40]. Such detection of a declining egg excretion intensity may have been prevented in the present study by the generally low liver fluke EPGs with a maximum of 2.5 across all parities.

The probability of rumen fluke egg excretion varied across parity numbers. Previous research has provided contradictory results, as Arias et al. [13] and Toolan et al. [12] found no association between rumen fluke prevalence and age, whereas other studies reported an increasing prevalence with age [9–11]. Rumen fluke EPGs significantly increased with increasing parity number. Similar observations were made by Ferreras et al. [10] and Bellet et al. [43], who observed higher worm burdens in older cattle, and by Teschner et al. [44], who detected higher egg counts in older dairy cows. Adult rumen flukes are known to invoke almost no immune response from their bovine host [45, 46] and are presumed to have a long lifespan. In addition, most anthelmintics used for treatment of *F. hepatica* are not effective against rumen flukes [47]. This may result in an accumulation of the parasite and therefore an increased egg excretion with higher age.

Coccidian oocyst excretion decreased in both probability and intensity after the first parity. This is in line with previous studies reporting a lower prevalence and lower OPGs in older cows, as *Eimeria* spp. are typically observed in calves and heifers, while adult cows are protected by immunity [2, 5]. However, as typical for parasites, the immunity is not sterile, i.e. protects against high burdens or disease, but does not prevent oocyst excretion, which explains why older cows may still excrete low numbers of oocysts [48].

The lactation stage significantly influenced the excretion probability of strongyle eggs. Higher excretion rates were observed in early lactation, with a subsequent decline as DIM increased. The peripartum and early lactation period has been associated with a higher frequency of numerous metabolic and infectious diseases in dairy cows [14, 16, 17]. While the exact mechanisms are still not fully understood, it is hypothesised that the negative energy balance during this period infers with the immune system [49], as it results in elevated ketone body concentrations, which have been shown to reduce the phagocytic and chemotactic capacity of immune cells, the number of blood leukocytes and cytokine production [50]. Additionally, amino acids are used for glucose synthesis, reducing the availability of metabolisable proteins, which are required for many immune components such as immunoglobulins, cytokines and mast cell proteases [51]. Hepatic lipidosis, resulting from rapid fatty acid mobilisation, may further impair immune responses, as fatty livers have been associated with reduced neutrophil function and increased severity of infectious diseases in dairy cows [52, 53]. In contrast to strongyles, the lactation stage had no significant effect on the egg/oocyst excretion probability of *F. hepatica*, rumen flukes and coccidia, suggesting that these infections may be less affected by metabolic changes during lactation than strongyle infections. For none of the endoparasites tested, excretion intensity was significantly affected by the lactation stage, contrary to previous observations of higher strongyle EPGs in early lactation [6, 18].

It has been hypothesised that endoparasite co-infections influence each other [19]. An increased co-occurrence of two endoparasite taxa may result from a suppression of the host’s immune response, which consequently increases the probability of infection with another endoparasite [20, 22]. However, influences may also be antagonistic, e.g. due to a competition for resources [20]. It is reasonable that parasites residing in the same organ are more closely linked owing to shared location and resources. Accordingly, the present study found significant interactions of co-infections between strongyles and coccidia, which both reside in the intestine. Coccidian co-infections significantly increased the probability of strongyle egg excretion, and conversely, strongyle co-infections raised the probability of coccidian oocyst excretion. Moreover, a coccidian co-infection was associated with higher strongyle EPGs but not vice versa. A positive correlation between infections with strongyle and coccidia has been reported in previous studies [20, 22, 23, 54]. Besides the age of the host, several other potential reasons, such as the direct life cycle of both strongyle and coccidia and their similar habitat, have been discussed for this correlation [23]. Additionally, potential synergistic effects between strongyles and coccidia within the host have been proposed [19, 54]. Gorsich et al. [20] suggested that the host’s immune system may have difficulty in responding simultaneously to extracellular (strongyles) and intracellular (coccidia) gastrointestinal parasites, as they provoke opposing immune responses. In this context, helminth infections have been shown to increase microparasite density by suppressing the inflammatory cytokine interferon-γ in mice [21]. However, co-infections with *F. hepatica* or rumen flukes had no significant effect on coccidian oocyst excretion in the present study.

The absence of significant interactions between coccidia and liver flukes, as well as between strongyles and flukes, aligns with previous reports [22, 23, 43, 55], although several studies observed a positive association between strongyles and *F. hepatica* [24, 25]. Furthermore, significant effects of fluke co-infections on egg excretion were not identified in the present study, although a positive association of liver fluke and rumen fluke co-infections was observed in previous studies [22, 24, 26, 43]. The varying results may be caused by different statistical methods or other differences in study design. Most of the studies mentioned above only calculated simple correlations without correcting for other potential influences. By contrast, the present study used a multivariate approach, incorporating additional influencing factors in statistical models. The rumen fluke hurdle model revealed that cows co-infected with coccidia had significantly lower rumen fluke EPGs. This may indicate a possible antagonistic effect, or it may be a remnant of the opposite patterns associated with age, namely the increase in rumen fluke EPG and the decrease in coccidia OPG with age. Thus, interaction effects between the co-infection status and parity number for each endoparasite taxa were tested, but the inclusion of interaction terms resulted in poorer model fits, i.e. larger AIC values, or the models did not converge.

It was also not possible to model the effect of multiple (double or triple) co-infections, because sample sizes in the respective groups were too small (cf. Table 2).

The generally high frequency of strongyle infections at both herd and individual level observed in this study is comparable to previous studies in European cattle [2, 36, 56]. The high percentage of *F. hepatica*-positive herds can be attributed to the preselection of farms on the basis of reported liver fluke infections. Nevertheless, the mean liver fluke in-herd prevalence was not higher than in studies without preselection [27, 57]. The high percentage of rumen fluke-positive herds and individual cows may also be affected by preselection, as the rumen fluke *C. daubneyi* develops in the same intermediate snail host as *F. hepatica*. However, the frequent occurrence of rumen fluke infections could also be due to the overall rapidly increasing prevalence in Europe [47]. The detection frequency of coccidian oocysts is consistent with previous reports in adult cattle [2, 22, 58]. The same applies to the less frequent occurrence of *Moniezia* spp., *Trichuris* spp. and *Capillaria* spp. [2, 36, 59]. The absence of *D. viviparus* larvae in the sampled cows was not unexpected, as lungworm infections in German dairy herds have been detected rather rarely in recent times [25].

The development of statistical models based on egg or oocyst excretion data is always challenging due to the high degree of variance, which does not follow a Gaussian distribution [60]. This issue was also encountered in the present study. While binary models (egg excretion yes/no) consistently provided good model fits, modelling the egg count data proved to be more difficult. Hurdle models, which are often used for datasets with an excess of zero values and are therefore well-suited for egg count data, ultimately provided the most successful approach [61, 62]. Their structure allowed us to distinguish whether factors affected the probability and/or the intensity (i.e., EPG/OPG values) of egg/oocyst excretion. However, the difficulty of fitting models for EPG/OPG values, particularly for rumen fluke EPGs which showed the highest variance, should be taken into account when interpreting the results of the count models. Another potential statistical bias was the uneven distribution of sample sizes between parity numbers, as the number of cows in parities 4 to ≥ 6 was lower than in parities 1–3 owing to the short productive lifespan of dairy cows [39]. Furthermore, the models cannot account for all the variables that may potentially influence endoparasite infections. Important influencing factors are the time spent on pasture and anthelmintic treatment of individual cows. Unfortunately, information on these factors was not available, which is a notable limitation of the current investigation. Future studies would benefit from access to individual treatment and pasturing data for such analyses.

## Conclusions

The results of this study showed a significant impact of individual host factors and co-infections on the prevalence and excretion intensity of endoparasite infections in dairy cows. Strongyle egg and coccidian oocyst excretion was more common in younger cows, whereas older cows were more likely to excrete *F. hepatica* eggs and had higher rumen fluke egg counts. Moreover, the lactation stage influenced strongyle egg excretion, with cows in early lactation being more likely to excrete strongyle eggs. A positive association between strongyle egg and coccidian oocyst excretion was observed, with co-infected cows showing higher excretion probabilities for both taxa as well as higher strongyle egg counts. While the increased occurrence of strongyles and coccidia in younger animals is consistent with established epidemiological patterns, the higher excretion of liver and rumen fluke eggs in older cows highlights their particular role in pasture contamination and the spread of fluke infections. Therefore, herd-level fluke monitoring could benefit from targeting cows in higher parities for routine faecal examinations.

## Supplementary Information

Additional file 1.

## Data Availability

Data supporting the findings of this study are available within the article and its additional files.

## Abbreviations

DIM: Days in milk
EPG: Eggs per gram of faeces
OPG: Oocysts per gram of faeces
HF: Holstein Friesian
AIC: Akaike information criterion
