# Supplementary material for: Individual host factors and co-infections affect the probability and excretion intensity of endoparasite infections in dairy cows
Source: Parasit Vectors. 2025 Jul 30;18:311. doi: 10.1186/s13071-025-06974-x (PMC12309137; doi:10.1186/s13071-025-06974-x)
Supplement: Supplementary file 1 — Additional file 1. [file 13071_2025_6974_MOESM1_ESM.docx]

**Additional file 1: Table S1.** Strongyle model: Results of the hurdle model investigating the influence of parity number, lactation stage and endoparasite coinfections on the probability and intensity of strongyle egg excretion in German dairy cows. Farm and cattle breed were included as random factors. Variables for parities 2 to ≥6 are to be interpreted in relation to parity 1.

|  | Estimate | SE | z-value | *P*-value |
| --- | --- | --- | --- | --- |
| **Binary model (excretion probability)** |  |  |  |  |
| Intercept | 0.17 | 0.34 | 0.50 | 0.617 |
| Parity 2 | -0.38 | 0.19 | -2.07 | 0.038* |
| Parity 3 | -0.73 | 0.21 | -3.56 | <0.001* |
| Parity 4 | -0.64 | 0.25 | -2.59 | 0.010* |
| Parity 5 | -0.24 | 0.28 | -0.86 | 0.390 |
| Parity ≥6 | 0.08 | 0.27 | 0.31 | 0.754 |
| Days in milk | -0.01 | 0.00 | -2.09 | 0.037* |
| Coinfection with *F. hepatica* | -0.03 | 0.24 | -0.13 | 0.900 |
| Coinfection with rumen flukes | 0.40 | 0.25 | 1.61 | 0.109 |
| Coinfection with coccidia | 0.47 | 0.18 | 2.54 | 0.011* |
| **Count model (excretion intensity)** |  |  |  |  |
| Intercept | 1.67 | 0.17 | 9.90 | <0.001* |
| Parity 2 | -0.34 | 0.20 | -1.69 | 0.092 |
| Parity 3 | -0.25 | 0.21 | -1.18 | 0.237 |
| Parity 4 | -0.26 | 0.26 | -1.03 | 0.303 |
| Parity 5 | -0.22 | 0.27 | -0.84 | 0.403 |
| Parity ≥6 | -0.41 | 0.25 | -1.63 | 0.104 |
| Days in milk | 0.00 | 0.00 | -0.40 | 0.690 |
| Coinfection with *F. hepatica* | 0.09 | 0.20 | 0.45 | 0.654 |
| Coinfection with rumen flukes | 0.13 | 0-16 | 0.77 | 0.442 |
| Coinfection with coccidia | 0.37 | 0.14 | 2.64 | 0.008* |

**P-*value≤0.05

SE=standard error

The full model was significantly different from a null model containing only the random factors (χ2=50.65, df = 25, deviance = 4238.20, *P*<0.001).

**Additional file 1: Table S2.** *Fasciola hepatica* model: Results of the hurdle model investigating the influence of parity number, lactation stage and endoparasite coinfections on the probability and intensity of *F. hepatica* egg excretion in German dairy cows. Farm and cattle breed were included as random factors. Variables for parities 2 to ≥6 are to be interpreted in relation to parity 1.

|  | Estimate | SE | z-value | *P*-value |
| --- | --- | --- | --- | --- |
| **Binary model (excretion probability)** |  |  |  |  |
| Intercept | -3.26 | 0.43 | -7.62 | <0.001* |
| Parity 2 | 0.50 | 0.35 | 1.41 | 0.159 |
| Parity 3 | 0.92 | 0.35 | 2.60 | 0.009* |
| Parity 4 | 1.08 | 0.37 | 2.90 | 0.004* |
| Parity 5 | 0.17 | 0.52 | 0.32 | 0.749 |
| Parity ≥6 | 1.27 | 0.39 | 3.29 | <0.001* |
| Days in milk | 0.00 | 0.00 | 1.21 | 0.226 |
| Coinfection with strongyles | -0.05 | 0.24 | -0.20 | 0.843 |
| Coinfection with rumen flukes | 0.61 | 0.24 | 1.84 | 0.067 |
| Coinfection with coccidia | -0.08 | 0.29 | -0.27 | 0.785 |
| **Count model (excretion intensity)** |  |  |  |  |
| Intercept | -0.81 | 1.23 | -0.66 | 0.511 |
| Parity 2 | 0.94 | 0.69 | 1.37 | 0.172 |
| Parity 3 | -0.20 | 0.62 | -0.33 | 0.744 |
| Parity 4 | 0.59 | 0.63 | 0.94 | 0.349 |
| Parity 5 | 0.67 | 0.88 | 0.94 | 0.450 |
| Parity ≥6 | 0.14 | 0.62 | 0.22 | 0.829 |
| Days in milk | 0.00 | 0.00 | 0.51 | 0.611 |
| Coinfection with strongyles | 0.37 | 0.47 | 0.78 | 0.433 |
| Coinfection with rumen flukes | -0.63 | 0.58 | -1.08 | 0.279 |
| Coinfection with coccidia | -0.68 | 0.51 | -1.33 | 0.184 |

*=*P-*value≤0.05

SE=standard error

The full model was significantly different from a null model containing only the random factors (χ2=33.21, df = 18, deviance = 996.84, *P*=0.016).

**Additional file 1: Table S3.** Rumen fluke model: Results of the hurdle model investigating the influence of parity number, lactation stage and endoparasite coinfections on the probability and intensity of rumen fluke egg excretion in German dairy cows. Farm and cattle breed were included as random factors. Variables for parities 2 to ≥6 are to be interpreted in relation to parity 1.

|  | Estimate | SE | z-value | *P*-value |
| --- | --- | --- | --- | --- |
| **Binary model (excretion probability)** |  |  |  |  |
| Intercept | -1.03 | 0.84 | -1.22 | 0.223 |
| Parity 2 | -0.03 | 0.36 | -0.07 | 0.941 |
| Parity 3 | 1.12 | 0.38 | 2.92 | 0.004* |
| Parity 4 | 0.19 | 0.45 | 0.41 | 0.681 |
| Parity 5 | 1.11 | 0.51 | 2.18 | 0.029* |
| Parity ≥6 | 0.09 | 0.44 | 0.21 | 0.832 |
| Days in milk | 0.00 | 0.00 | 0.74 | 0.460 |
| Coinfection with strongyles | 0.41 | 0.28 | 1.44 | 0.151 |
| Coinfection with *F. hepatica* | 0.46 | 0.38 | 1.21 | 0.226 |
| Coinfection with coccidia | 0.43 | 0.32 | 1.34 | 0.180 |
| **Count model (excretion intensity)** |  |  |  |  |
| Intercept | 1.50 | 1.54 | 0.97 | 0.331 |
| Parity 2 | 0.16 | 0.26 | 0.61 | 0.542 |
| Parity 3 | 0.64 | 0.24 | 2.61 | 0.009* |
| Parity 4 | 0.61 | 0.24 | 2.50 | 0.013* |
| Parity 5 | 0.96 | 0.27 | 3.56 | <0.001* |
| Parity ≥6 | 1.12 | 0.25 | 4.54 | <0.001* |
| Days in milk | 0.00 | 0.00 | -0.25 | 0.806 |
| Coinfection with strongyles | -0.12 | 0.15 | -0.79 | 0.429 |
| Coinfection with *F. hepatica* | 0.05 | 0.21 | 0.23 | 0.817 |
| Coinfection with coccidia | -0.52 | 0.21 | -2.45 | 0.014* |

*=*P-*value≤0.05

SE=standard error

The full model was significantly different from a null model containing only the random factors (χ2=58.93, df = 25, deviance = 3634.80, *P*<0.001).

**Additional file 1: Table S4.** Coccidia model: Results of the hurdle model investigating the influence of parity number, lactation stage and endoparasite coinfections on the probability and intensity of coccidian oocyst excretion in German dairy cows. Farm and cattle breed were included as random factors. Variables for parities 2 to ≥6 are to be interpreted in relation to parity 1.

|  | Estimate | SE | z-value | *P*-value |
| --- | --- | --- | --- | --- |
| **Binary model (excretion probability)** |  |  |  |  |
| Intercept | -1.61 | 0.29 | -5.54 | <0.001* |
| Parity 2 | -0.38 | 0.22 | -1.76 | 0.079 |
| Parity 3 | -1.15 | 0.28 | -4.10 | <0.001* |
| Parity 4 | -1.16 | 0.34 | -3.40 | 0.001* |
| Parity 5 | -0.71 | 0.36 | -2.00 | 0.049* |
| Parity ≥6 | -1.19 | 0.36 | -3.30 | 0.001* |
| Days in milk | 0.00 | 0.00 | 1.66 | 0.098 |
| Coinfection with strongyles | 0.49 | 0.18 | 2.67 | 0.008* |
| Coinfection with *F. hepatica* | -0.11 | 0.28 | -0.38 | 0.704 |
| Coinfection with rumen flukes | 0.46 | 0.27 | 1.68 | 0.092 |
| **Count model (excretion intensity)** |  |  |  |  |
| Intercept | 2.04 | 0.39 | 5.27 | <0.001* |
| Parity 2 | -0.48 | 0.24 | -2.03 | 0.043* |
| Parity 3 | -0.63 | 0.32 | -1.99 | 0.046* |
| Parity 4 | -0.76 | 0.38 | -1.99 | 0.046* |
| Parity 5 | -0.98 | 0.42 | -2.34 | 0.019* |
| Parity ≥6 | -0.76 | 0.41 | -1.88 | 0.061 |
| Days in milk | 0.00 | 0.00 | -1.91 | 0.056 |
| Coinfection with strongyles | -0.06 | 0.19 | -0.32 | 0.751 |
| Coinfection with *F. hepatica* | -0.34 | 0.31 | -1.07 | 0.286 |
| Coinfection with rumen flukes | -0.17 | 0.27 | -0.62 | 0.537 |

*=*P-*value≤0.05

SE=standard error

The full model was significantly different from a null model containing only the random factors (χ2=65.45, df = 25, deviance = 2095.80, *P*<0.001).
